# Cephalic Tetanus Presenting as Peripheral Facial Palsy: A Case Report

**DOI:** 10.7759/cureus.37116

**Published:** 2023-04-04

**Authors:** Reda Hamdi, Mohamed Afellah, Mohamed Ridal, Mohamed Amine Elalami

**Affiliations:** 1 Department of Otolaryngology, University Hospital Center Hassan II, Fez, MAR; 2 Department of Otorhinolaryngology, University Hospital Center Hassan II, Fez, MAR

**Keywords:** case report, tetanus, cephalic revelation, facial nerve, facial palsy

## Abstract

Tetanus is a serious disease that has become rare in countries that adopt an effective and sustainable vaccination strategy; however, tetanus remains a fairly common problem in developing countries. The diagnosis of tetanus is fairly easy. However, its cephalic form is a rare but potentially life-threatening neurological condition caused by the bacteria *Clostridium tetani*, which can result in symptoms such as spasms, rigidity, and paralysis of various muscles and nerves in the head and neck region.

This article is about a 43-year-old man who had presumed an idiopathic facial palsy at the beginning and who turned out after the evolution of the clinical picture that it is cephalic tetanus. In this article, we will see the subtleties and clinical elements that have allowed us to rectify the diagnosis. Peripheral facial palsy can be a presenting symptom of cephalic tetanus and should be considered in patients with a history of tetanus infection or exposure. Early recognition and prompt treatment of cephalic tetanus are crucial in preventing complications and improving outcomes. Treatment typically involves the administration of tetanus immunoglobulin and antibiotics, as well as supportive care for any associated symptoms or complications.

## Introduction

Facial palsy is a condition that affects the facial nerve and causes weakness or total paralysis of the facial muscles that control expression [1]. It can be divided into the following two types depending on the location of the insult: central facial palsy (due to supranuclear damage) and peripheral facial palsy (due to infranuclear damage). Bell’s palsy is the most common cause of peripheral facial palsy, which has an unclear etiology but is thought to be caused by herpes simplex virus activity. Other causes of peripheral facial palsy include trauma, particularly fractures of the petrous bone; Lyme disease; complicated middle ear otitis; diabetic neuropathy; a facial nerve tumor; Guillain-Barré syndrome; leprosy; multiple sclerosis; polyarteritis nodosa; sarcoidosis; stroke; and syphilis [2].

Facial palsy due to cephalic tetanus is a rare but serious complication of tetanus. Cephalic tetanus occurs when the infection affects the cranial nerves. The most frequently involved cranial nerve is the seventh. It accounts for 1% to 3% of the total number of reported cases of tetanus and has a mortality rate of 15% to 30%. The incubation period is 1 to 14 days, and approximately two-thirds of cases progress to generalized tetanus [3]. Cephalic tetanus is defined as a combination of trismus and paralysis of one or more cranial nerves. Although the facial nerve is involved in most cases of cephalic tetanus, cephalic tetanus is rarely considered to be a cause of facial nerve palsy. The mechanism of the paralysis is not completely understood. Treatment involves debridement of wounds, administration of penicillin and tetanus immune globulin, aggressive supportive care, and initiation of active immunization. This type of tetanus typically occurs following facial injuries or wounds and can cause weakness or total paralysis of the facial muscles that control expression. The diagnosis of cephalic tetanus can be difficult, as it may not present with the classic symptoms of tetanus, such as muscle stiffness and spasms [4].

## Case presentation

A 43-year-old farmer was consulted in the emergency room of the Hassan II University Hospital in Fez for sudden difficulty closing the left eye, saliva leakage, hypersensitivity to sound and light, and weakness of the left side of his face.

**Figure 1:**
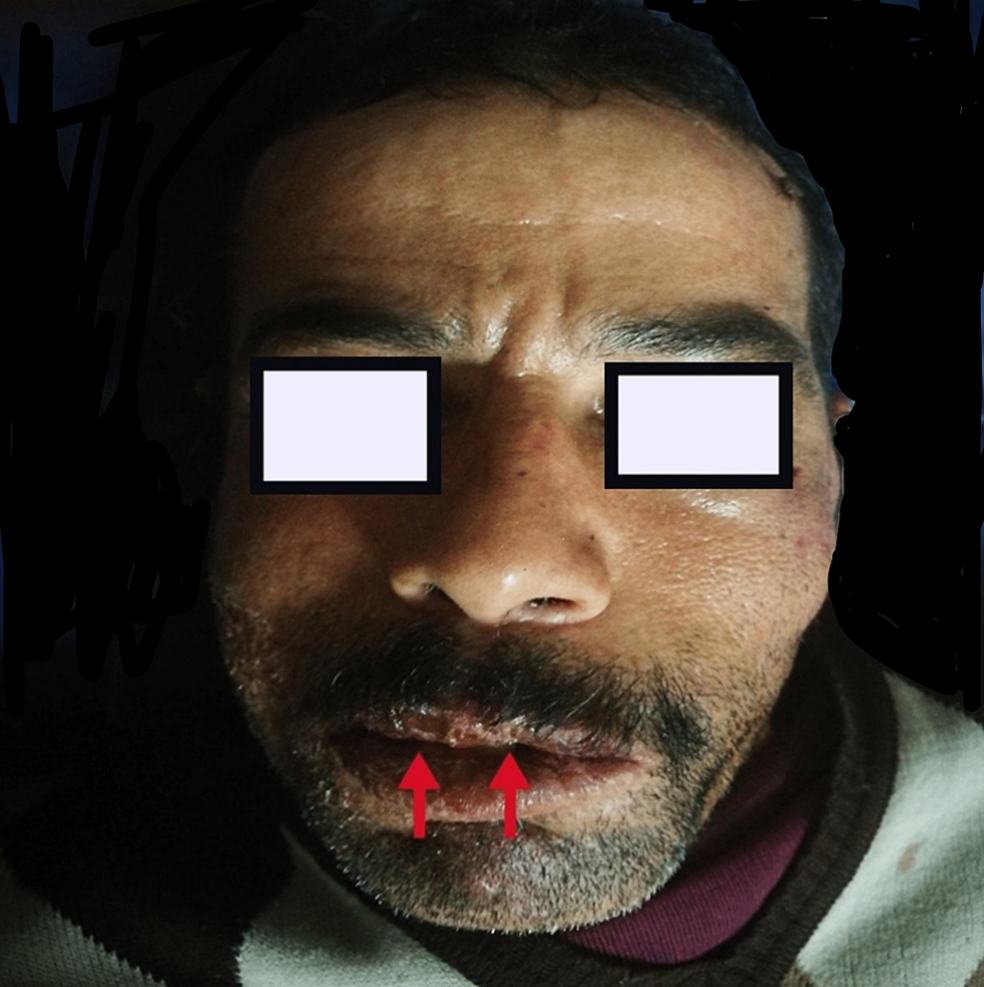
Patient at time of diagnosis: left facial palsy at rest showing a disappearance of nasolabial folds and frontal wrinkles, with a deviation of the mouth towards the right side, and erosive cheilitis with lips fissures highlighted with red arrows.

**Figure 2:**
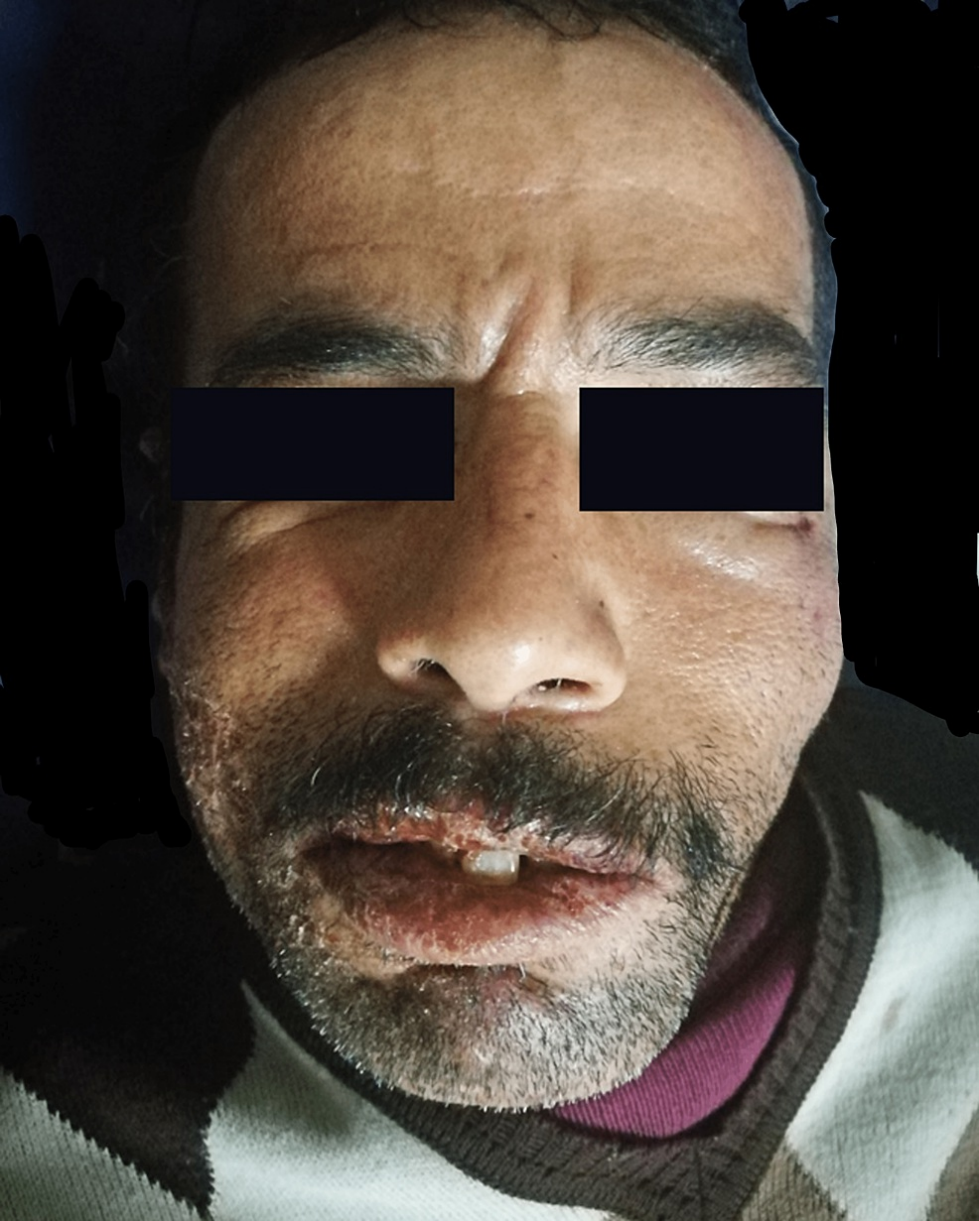
Patient at time of diagnosis: left facial palsy and trismus.

The physical examination found a drooping left eyebrow and a drooping corner of the mouth, a labial fissure, and muscle testing was rated 18 out of 30 and stage III, according to the House and Brackmann [5] grading system (Figure 2). History revealed no other abnormalities that could have caused FP. Erosive cheilitis with beads appeared 10 days earlier (Figure 1). This cheilitis was not the subject of local treatment. Otoscopy, facial tenderness, and general neurologic examination (especially of other cranial nerves) were normal. 

It wasn't necessary to practice an electrophysiologist’s test because of the peripheral character of the facial paralysis, its short duration of evolution, which did not exceed four days, and the absence of traumatic cause to give an idea of the possible interest of surgical exploration.

The patient was afebrile. The remainder of the somatic examination was unremarkable. The “idiopathic“ FP or Bell's palsy diagnosis was retained; oral antiviral drugs and corticosteroid therapy were prescribed. Four days later, FP worsened, and muscle testing was rated 10 out of 30. Bilateral trismus completed the picture with contractures of neck muscles.

**Figure 3:**
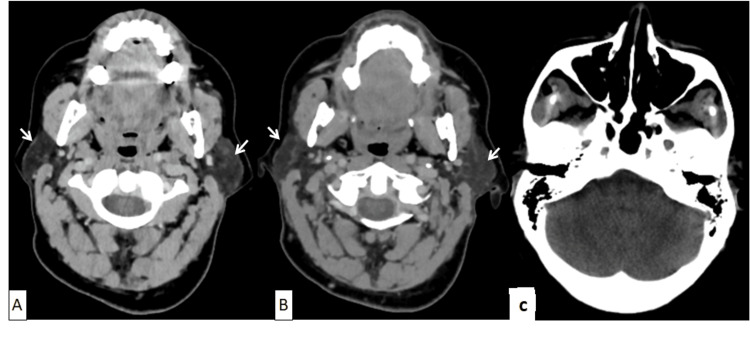
Axial scannographic sections at the craniocervical level: absence of abnormality of the cerebral parotids (Figure A) and at the level of the deep spaces of the face, which could possibly explain the trismus (Figure B) and posterior cerebral fossa with no abnormality (Figure C).

**Figure 4:**
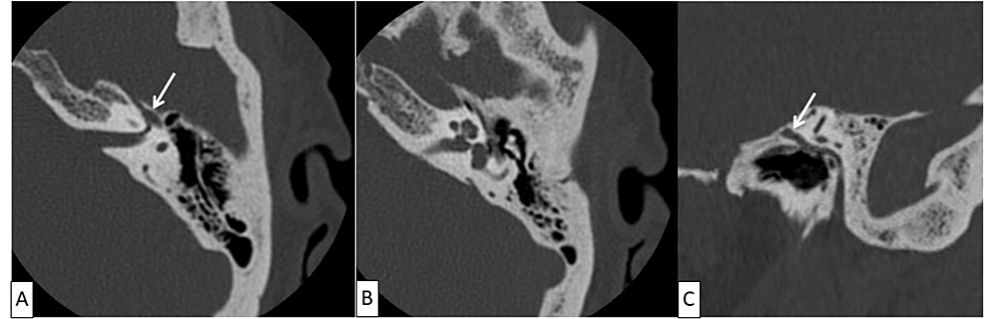
CT scan of the left petrous bone in axial sections: absence of anomalies in the left facial canal first portion and the geniculate ganglion (A), middle ear (B), and petrous bone and the second portion of the facial canal (C).

The mouth opening was one finger across. Dysphagia progressively worsened and deteriorated without a febrile syndrome. The other cranial nerves were intact, and pharyngolaryngeal nasofibroscopy was normal. The deep spaces of the face and facial nerve appeared normal on the CT scan (Figures 3, 4).

These clinical and anamnestic data raised fears of cephalic tetanus, especially because the patient was not vaccinated against tetanus and the labial fissures were at any time subject to care, and then with the worsening of the clinical presentation and the appearance of trismus.

The patient was transferred to an intensive care unit and treated with penicillin G (6,000,000 IU/day parenterally for 12 days), a dose of 3,500 IU of tetanus serum by subcutaneous injection, and 40 mg/day of diazepam by intravenous infusion for five days followed by oral administration. The lip wound was debrided with oxygenated water. Trismus improved; the mouth opening normalized after 10 days, and FP declined (muscle testing changed from 10 out of 30 to 18 out of 30).

The patient could have been saved thanks to the attention paid to the change in the clinical presentation. After 10 days spent in the intensive care unit, the patient was transferred to the ENT department, where he spent a week. The evolution was favorable without a sequel, and the patient was discharged with strict vaccination recommendations.

## Discussion

This case of cephalic tetanus was characterized by peripheral facial palsy (FP), and trismus appeared only secondarily. The appearance of trismus refuted the initial diagnosis of idiopathic FP. This pathology has become rare owing to vaccination and preventive tetanus serotherapy in the event of a wound. It is becoming unrecognized, especially in localized forms (in particular cephalic) [6,7]. The gateway is a facial sore (even a puncture), chronic otitis media, or a dental or oral lesion [8]. In our case, it was an erosive cheilitis.

Similar to our case, where the facial nerve was the only facial nerve impacted, the facial nerve is the cranial nerve that is most frequently on the side of the entry point. Paralysis is of the peripheral type. Its physiopathology remains unclear; centripetally transported neurotoxins may cause damage to motor neurons in the nucleus of the facial nerve [9,10]. The diagnosis of so-called “rose” cephalic tetanus is clinical. It is more likely in the case of a facial sore. The clinical picture associate trismus and FP with or without oculomotor palsy. In some cases, FP can precede trismus, as we observed in our patient, which may delay diagnosis and treatment. Fortunately, in our case, the diagnosis could be rectified by a second consultation with the patient following the aggravation of the clinical picture and the appearance of the trismus, which allowed for the diagnosis of cephalic tetanus.

The treatment involves close supervision in a medical intensive care unit owing to the risk of transitioning to a generalized form, which occurs in two-thirds of cases, and isolation from light and noise. Myorelaxants are needed to fight muscle contraction and reduce pain [11,12]. Debridement of the gateway is essential. Oxygenated water is recommended because bacteria are strictly anaerobic [13].

Antibiotics (beta-lactams, tetracyclines, and metronidazole) limit commensal microbial overgrowth, which aggravates anaerobiosis. This anaerobic atmosphere promotes the release of toxic neurotropic by tetanus bacillus, which leaves its vegetative sporulated form [14].

Concerning the tetanus vaccine efficacy and effectiveness, the antibody concentration and avidity and the duration of protection depend on several factors, including the age of the vaccines and the number of intervals between vaccine doses. Three DTP doses in infancy will give 3-5 years’ protection; a further dose or booster (e.g., in early childhood) will protect adolescence; and one or two more booster(s) will induce immunity well through adulthood - 20-30 years has been suggested. Booster responses can still be elicited after intervals of 25-30 years, demonstrating the persistence of immunological memory [15].

## Conclusions

Facial palsy due to cephalic tetanus is a rare but serious complication of tetanus. Cephalic tetanus occurs when the infection affects the cranial nerves, including the facial nerve. This type of tetanus typically occurs following facial injuries or wounds and can cause weakness or total paralysis of the facial muscles that control expression. The diagnosis of cephalic tetanus can be difficult, as it may not present with the classic symptoms of tetanus, such as muscle stiffness and spasms. The facial nerve is the most affected cranial nerve, most often on the side of the entry point. Paralysis is of the peripheral type. Its physiopathology remains unclear; centripetally transported neurotoxins may cause damage to motor neurons in the nucleus of the facial nerve. The diagnosis of so-called “rose” cephalic tetanus is clinical. It is more likely in the case of a facial sore. The treatment involves close supervision in a medical intensive care unit owing to the risk of transitioning to a generalized form, which occurs in two-thirds of cases, and isolation from light and noise. Myorelaxants are needed to fight muscle contraction and reduce pain. Debridement of the gateway is essential. Oxygenated water is recommended because bacteria are strictly anaerobic. Antibiotics (beta-lactams, tetracyclines, and metronidazole) limit commensal microbial overgrowth, which aggravates anaerobiosis. This anaerobic atmosphere promotes the release of toxic neurotropic by tetanus bacillus, which leaves its vegetative sporulated form.
